# Group A Streptococcus Necrotizing Lymphadenitis: A Case Report

**DOI:** 10.7759/cureus.33699

**Published:** 2023-01-12

**Authors:** Nathalie De Paz, Arian Pupo, Lexi R Frankel, Summer L Roorda, Robert Hernandez

**Affiliations:** 1 Internal Medicine, Kendall Regional Medical Center, Kendall, USA; 2 Obstetrics and Gynecology, Nova Southeastern University Dr. Kiran C. Patel College of Allopathic Medicine, Davie, USA; 3 Infectious Disease, Kendall Regional Medical Center, Kendall, USA

**Keywords:** bacterial lymphadenitis, systemic lupus erythematosus, pathology, infectious disease, group a streptococcus, necrotizing lymphadenitis

## Abstract

Necrotizing lymphadenitis (NL) is a rare entity that can occur as a complication of bacterial cervical lymphadenitis (CL) and is characterized by unilateral or bilateral cervical lymphadenopathy. NL most commonly presents in females and most reports have been in Japan. In this case, we present a 37-year-old male with no significant past medical history who presented with an unusual presentation and clinical course of NL. Initial workup for Epstein-Barr Virus (EBV) and other infectious etiology was negative. Yet, aspiration later revealed Group A Streptococcus. When the patient's pain and swelling did not subside with the initial antibiotic and supportive treatment, the patient underwent a repeat aspiration and biopsy that revealed a necrotic mass or lymph node. NL is uncommon and rarely due to infectious etiology. However, this marks a case in which a Group A Streptococcus was shown to be associated with subsequent necrotic lymph nodes and should allow practitioners to further consider an infectious etiology in the differential diagnosis of NL.

## Introduction

Cervical lymphadenitis (CL) is characterized by enlargement of the unilateral or bilateral cervical lymph nodes and is caused mainly by S*taphylococcus aureus* and Group A beta-hemolytic *Streptococci *(GABHS) [1]. The vast majority of studies on CL have been on children, for which this disease is commonly a transient response to a benign local or generalized infection [1]. In adults, there are also various fungal, bacterial, aerobic, and anaerobic organisms that have been documented as culprits causing infectious CL [2].

Necrotizing lymphadenitis (NL) is a rare entity that can occur as a complication of bacterial lymphadenitis. More frequently, NL is associated with chronic inflammatory states and infections and less often associated with acute illnesses. NL is often misdiagnosed given its variable clinical presentation and limited literature on the disease [3]. While NL can occur following bacterial infection, it is more commonly a precursor manifestation of systemic lupus erythematosus (SLE) and has been known to mimic tuberculosis and lymphoma [4]. NL, even in the setting of bacterial infection, therefore requires regular follow-up after hospital discharge [3]. Other common causes of NL include Kikuchi-Fujimoto disease, acute Epstein-Barr Virus (EBV) infection, and viral upper respiratory diseases [4]. Most cases of NL are present in females below the age of 40 with acute or subacute onset of fever and unilateral cervical lymphadenopathy [5]. Other commonly presenting symptoms include leukopenia, fever, myalgias, dysphagia, chest pain, and abdominal pain. Less commonly, NL has presented with Raynaud’s phenomenon and alopecia [6].

In this report, we present a case of a 37-year-old male without a significant past medical history with NL presenting after streptococcus pyogenes lymphadenitis. The occurrence of NL in a male after GABHS lymphadenitis is an infrequent occurrence that has been documented scarcely elsewhere in the literature.

## Case presentation

This patient is a 37-year-old male with no significant past medical history who initially presented to the emergency department (ED) with one week of worsening neck pain and swelling, malaise, and subjective fevers. The patient first developed a sore throat, followed by bilateral neck pain and swelling. Over the next week, the pain and swelling on the right side of the neck improved while the left-sided swelling worsened. He denied trismus, drooling, and dysphonia.

**Figure 1:**
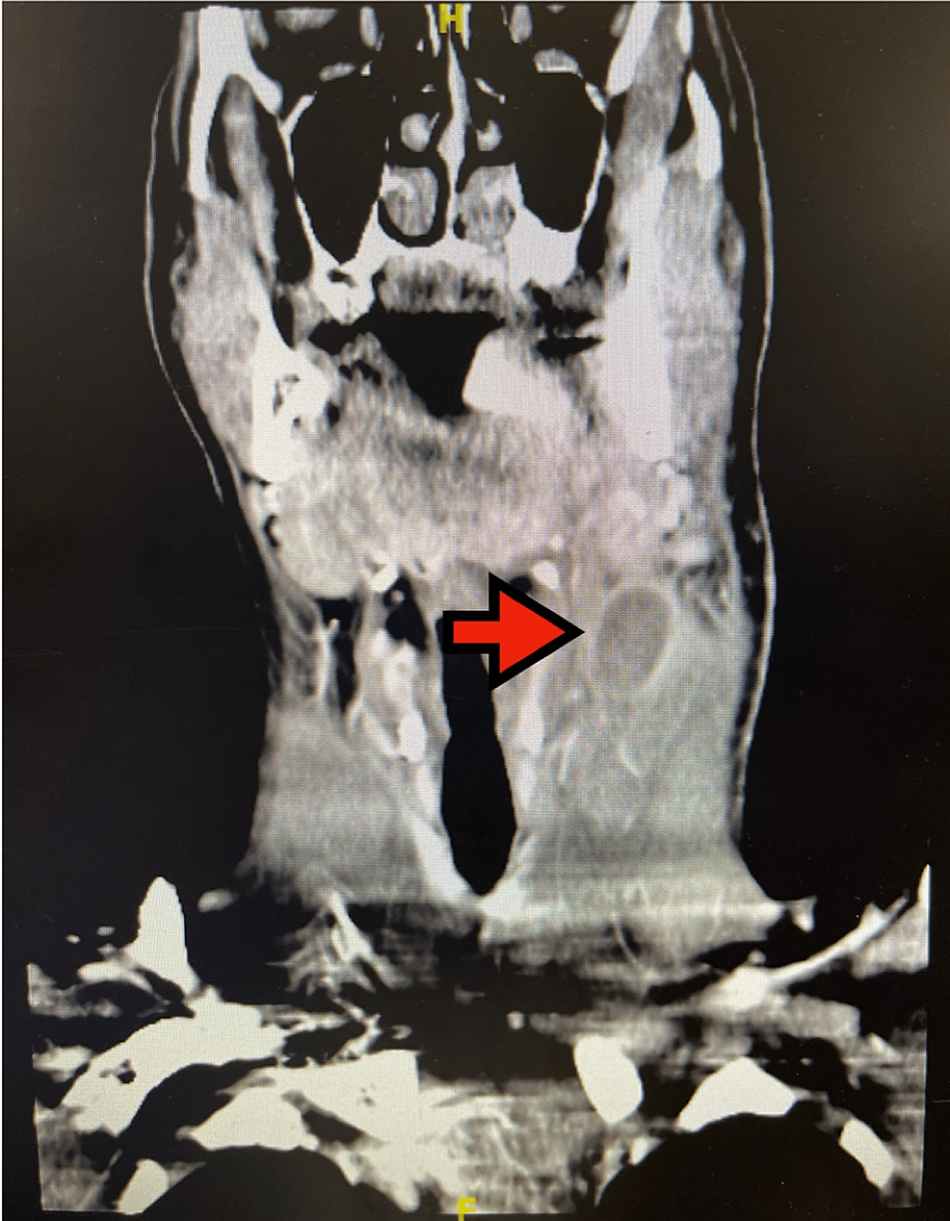
Frontal view CT scan. Red arrow demonstrates left neck mass, likely lymphatic in origin. CT: computed tomography

**Figure 2:**
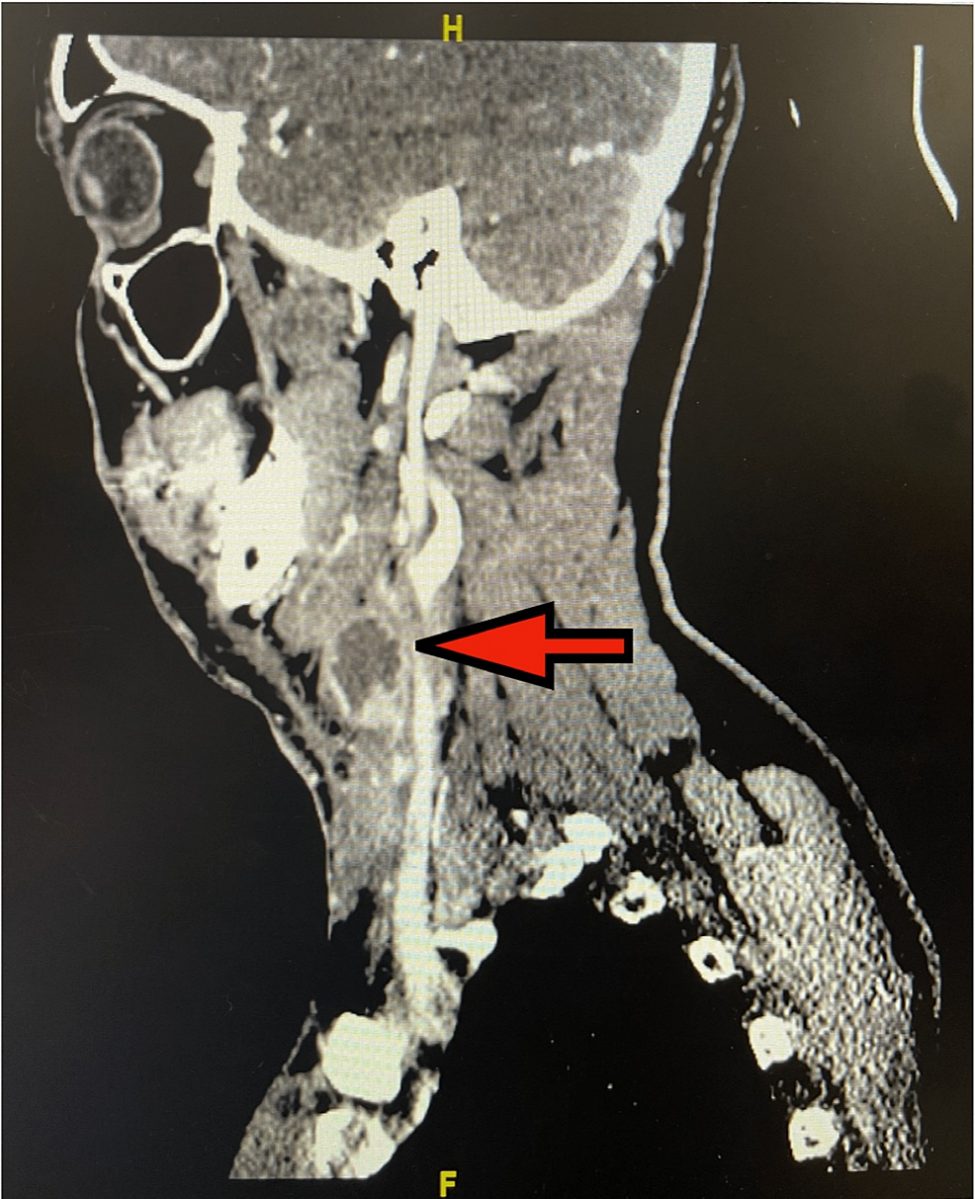
Sagittal view CT scan. Red arrow shows left neck mass, likely lymphatic in origin. CT: computed tomography

On physical exam, a left-sided cervical lymph node was exquisitely tender to palpation measuring 2 cm x 2 cm with a 3 cm surrounding area of erythema. No induration or fluctuance was noted. Initial labs revealed leukocytosis (21.4 K/uL) with neutrophilia and monocytosis. A computed tomography (CT) scan of the neck with contrast showed enlarged cervical lymph nodes scattered throughout the neck bilaterally and a 1.7 x 1.6 cm cystic focus within a cervical lymph node (Figure 1 and Figure 2).

CT of the abdomen and pelvis was unremarkable. The patient was then admitted and started on piperacillin-tazobactam for empiric treatment as well as dexamethasone to reduce neck swelling. Mononucleosis heterophile antibody test was negative, however, due to a high false negative rate in this test in the first week of infection, an EBV Immunoglobulin M and polymerase chain reaction were ordered. Additional serologies ordered included *Bartonella quintana*, SARS-COV-2, Cytomegalovirus, Human Immunodeficiency Virus, Influenza A and B, Toxoplasma, and Group A *Streptococcus*. The following day, the patient underwent aspiration of the cyst via interventional radiology with cultures taken. At this point, EBV Immunoglobulin M was negative and Toxoplasmosis Immunoglobulin M resulted and was negative. The additional serology and culture results were still pending. The patient reported noted some clinical improvement and decreased neck swelling and was discharged with doxycycline and amoxicillin-clavulanate.

Two days later, the patient returned to the ED with worsening left-sided neck pain and swelling. This same day, the aspirate from the previous visit was positive for Group A *Streptococcus* and the patient was started on ampicillin-sulbactam and doxycycline. Blood cultures at this point were negative. Repeat neck CT showed a suppurative necrotic lymph node versus abscess measuring up to 2.7 cm in the left submandibular space that had slightly increased in size since the last visit. Differential diagnosis based on imaging findings included a necrotic infected lymph node, necrotic metastasis, or an infected branchial cleft cyst. A branchial cleft cyst seemed unlikely in this patient based on the initial bilateral presentation and lack of cystic nature of the mass. However, otolaryngology and hematology-oncology were both consulted on the case. For diagnostic evaluation of possible lymphoproliferative process versus SLE, an immunologic panel consisting of serum immunoglobulin G, immunoglobulin A, immunoglobulin M, immunoglobulin E, and antineutrophil antibodies were ordered, all of which were within normal limits. Repeat ultrasound-guided fine needle aspiration/biopsy of the neck mass was subsequently performed. During the aspiration, no fluid could be obtained after multiple attempts suggesting a solid or necrotic mass or lymph node. At this point, the patient’s pain was well-managed and he was medically stable. He was discharged with instructions to follow up with otolaryngology, infectious disease, and his primary care physician. A biopsy later revealed lymphoid tissue with rare granulation tissue with acute and chronic inflammation without overexpression of a lymphoproliferative process or carcinoma further supporting the diagnosis of NL secondary to Group A *Streptococcus* infection.

## Discussion

The occurrence of NL in a male after GABHS lymphadenitis is exceedingly rare, making prompt diagnosis and treatment a clinical challenge [7]. While the majority of cases of NL have been reported in Japan, an increasing number of cases have been surfacing in the United States and Europe [7]. Previously reported cases have focused on its predilection for females in their twenties and thirties [7]. However, it is unknown why exactly this disease more commonly affects females. It is possible that the co-occurrence of NL with SLE, which is known to more commonly affect females, might be partly responsible for this pattern [8]. It is imperative to hold a high index of suspicion for all patients, not just females, who present with a neck mass, cervical lymphadenopathy, and common-cold symptoms.

NL has been documented as a presenting symptom or flare of SLE [8]. While this patient’s antinuclear antibody (ANA) was negative at the time of his hospital stay, it is vital to note that this patient was informed of the warning symptoms of autoimmune disease and to follow up with rheumatology for confirmation. A thorough history of neurologic, skin, and joint manifestations was also taken from the patient without any report of symptoms concerning autoimmune processes. Patients should be fully worked up to exclude underlying chronic disease and followed up closely to ensure full resolution of symptoms.

The scarcity of NL research, combined with its rarity, makes establishing an expected clinical course very difficult and controversial. Previous studies have noted that improvement in swelling and pain could be associated with the resolution of the disease and expected recovery [9]. However, in this case, the patient demonstrated an improvement in neck swelling and reported pain, however, presented back to the ED two days later with worsening symptoms. It should be noted that an initial improvement of symptoms does not exclude the diagnosis of NL. It may in fact present with a transient reduction in pain and swelling followed by acute clinical deterioration in a few days. Patients should be warned regarding this possibility to ensure prompt representation and treatment.

There are several diagnostic challenges of NL, which further add to the complexity of this disease. Diagnostic imaging usually shows enlarged cervical lymph nodes with hypervascular cortices and areas of necrosis [10]. The patient in this study did demonstrate these findings on imaging, however, the nonspecific nature of imaging findings further complicated our diagnostic pathway. The diagnosis of NL is confirmed with pathology, not radiology alone [10]. Histopathologic findings specific to NL include paracortical coagulative necrosis with karyorrhectic debris, abundant histiocytes, and the absence of neutrophils [10]. However, the initial biopsy on the patient in this case report was unsuccessful at removing any tissue. A subsequent biopsy later revealed lymphoid tissue with rare granulation tissue with acute and chronic inflammation without overexpression of a lymphoproliferative process or carcinoma. While these findings supported the diagnosis of NL secondary to Group A *Streptococcus* infection, again, they were not specific. This nonspecific nature of GABHS NL in diagnosis, radiology, and pathology is yet another reason to keep a high index of suspicion for this disease.

## Conclusions

NL occurring in a male, especially without a history of autoimmune disease, is an extremely rare phenomenon without many clinical recommendations from prior literature. This disease should be further studied in the male population to understand how the clinical course may vary in presentation, time course, and recovery. Additionally, providers should broaden their differential to include NL when presented with a patient endorsing a neck mass with cervical lymphadenopathy and signs of infection.
